# Increased expression of matrix metalloproteinase 3 can be attenuated by inhibition of microRNA-155 in cultured human astrocytes

**DOI:** 10.1186/s12974-018-1245-y

**Published:** 2018-07-21

**Authors:** Anatoly Korotkov, Diede W. M. Broekaart, Jackelien van Scheppingen, Jasper J. Anink, Johannes C. Baayen, Sander Idema, Jan A. Gorter, Eleonora Aronica, Erwin A. van Vliet

**Affiliations:** 1 Department of (Neuro) Pathology, Amsterdam UMC, University of Amsterdam, Meibergdreef 9, 1105 AZ Amsterdam, The Netherlands; 2 Department of Neurosurgery, Amsterdam UMC, Vrije Universiteit Amsterdam, De Boelelaan 1117, 1081 HV, Amsterdam, The Netherlands; 3 Swammerdam Institute for Life Sciences, Center for Neuroscience, University of Amsterdam, Science Park 904, 1098 XH, Amsterdam, The Netherlands; 4 Stichting Epilepsie Instellingen Nederland (SEIN), Heemstede, The Netherlands

**Keywords:** Epileptogenesis, Temporal lobe epilepsy, Extracellular matrix, MiRNA-155, MMP3

## Abstract

**Background:**

Temporal lobe epilepsy (TLE) is a chronic neurological disease, in which about 30% of patients cannot be treated adequately with anti-epileptic drugs. Brain inflammation and remodeling of the extracellular matrix (ECM) seem to play a major role in TLE. Matrix metalloproteinases (MMPs) are proteolytic enzymes largely responsible for the remodeling of the ECM. The inhibition of MMPs has been suggested as a novel therapy for epilepsy; however, available MMP inhibitors lack specificity and cause serious side effects. We studied whether MMPs could be modulated via microRNAs (miRNAs). Several miRNAs mediate inflammatory responses in the brain, which are known to control MMP expression. The aim of this study was to investigate whether an increased expression of MMPs after interleukin-1β (IL-1β) stimulation can be attenuated by inhibition of the inflammation-associated miR-155.

**Methods:**

We investigated the expression of MMP2, MMP3, MMP9, and MMP14 in cultured human fetal astrocytes after stimulation with the pro-inflammatory cytokine IL-1β. The cells were transfected with miR-155 antagomiR, and the effect on MMP3 expression was investigated using real-time quantitative PCR and Western blotting. Furthermore, we characterized MMP3 and miR-155 expression in brain tissue of TLE patients with hippocampal sclerosis (TLE-HS) and during epileptogenesis in a rat TLE model.

**Results:**

Inhibition of miR-155 by the antagomiR attenuated MMP3 overexpression after IL-1β stimulation in astrocytes. Increased expression of MMP3 and miR-155 was also evident in the hippocampus of TLE-HS patients and throughout epileptogenesis in the rat TLE model.

**Conclusions:**

Our experiments showed that MMP3 is dynamically regulated by seizures as shown by increased expression in TLE tissue and during different phases of epileptogenesis in the rat TLE model. MMP3 can be induced by the pro-inflammatory cytokine IL-1β and is regulated by miR-155, suggesting a possible strategy to prevent epilepsy via reduction of inflammation.

**Electronic supplementary material:**

The online version of this article (10.1186/s12974-018-1245-y) contains supplementary material, which is available to authorized users.

## Background

Epilepsy is a common neurological disease characterized by spontaneous recurrent seizures, affecting more than 50 million people worldwide. It is defined by any of the following conditions: (1) at least two unprovoked (or reflex) seizures occurring >  24 h apart. (2) One unprovoked (or reflex) seizure and a probability of further seizures similar to the general recurrence risk (at least 60%) after two unprovoked seizures, occurring over the next 10 years. (3) Diagnosis of an epilepsy syndrome [1]. Temporal lobe epilepsy (TLE) is one of the most common forms of focal epilepsy in adults [2, 3], which is frequently associated with hippocampal sclerosis [4]. The neuropathological processes associated with human TLE include neuronal loss, aberrant axonal growth and neurogenesis in the hippocampus, neuroinflammation, gliosis, reorganization of the extracellular matrix (ECM), and blood–brain barrier (BBB) dysfunction [5–9].

Matrix metalloproteinases (MMPs) constitute a class of proteases responsible for the remodeling of the ECM. Under normal physiological conditions various MMPs are involved in ECM homeostasis and synaptic plasticity in the brain [10–13]. Under pathological conditions, MMPs can be activated by a variety of stimuli including pro-inflammatory cytokines [14]. The pro-inflammatory cytokine IL-1β can be produced in the central nervous system (CNS) by activated astrocytes in response to tissue damage, increased neuronal activity or cellular stress [15–18]. Astrocytes contribute to chronic neuroinflammation in epilepsy not only as the major source of IL-1β [17], but also due to their role in K^+^ buffering, uptake of extracellular glutamate, glutamine supply for presynaptic terminals [19] as well as the ability to control synaptogenesis [20]. The deregulation of MMP expression and activity has been also associated with TLE, where it may contribute to altered neuronal excitability, acute and chronic neuroinflammation, neurodegeneration, gliosis and a compromised BBB [14, 21–25]. In the electrical post-status epilepticus (SE) rat TLE model, a large-scale transcriptome study revealed that the expression of MMP2, MMP3, MMP9 and MMP14 in the brain was increased and dynamically regulated at different stages of epileptogenesis [26]. The role of MMP9 in epileptogenesis has been extensively studied in various animal models [22] with the focus on the modulation of synaptic plasticity associated with seizures [27]. MMP3 has also been implicated in neurodegenerative disorders [28–31] and shown to contribute to the increased BBB permeability and apoptosis [32, 33]. Increased expression of MMP3 expression was previously demonstrated in the hippocampus after kainic acid-induced seizures in mice [34] and after pilocarpine-induced status epilepticus in rats [35]. In summary, accumulating evidence indicates that increased expression and/or activity of MMPs after an insult can contribute to epileptogenesis. Therefore, reducing MMP expression or activity has been suggested as a strategy for the prevention and/or modulation of epileptogenesis.

The increased gene expression under pathological conditions can be modulated by miRNAs. miRNAs are small non-coding RNAs capable of regulating target gene expression at post-transcriptional level [36–38]. miRNAs have been shown to be involved in the regulation of various biological processes within the CNS [39, 40] and have been implicated in neurological disorders, such as epilepsy [41, 42]. miRNAs can regulate gene expression directly through complementary binding to multiple messenger RNA (mRNA) transcripts and indirectly through modulating intracellular signaling pathways associated with the target genes. Several miRNAs have been shown to mediate inflammation in the brain [40]. This includes miR-146a, which inhibits inflammation in astrocytes [43–45]. Another inflammatory miRNA, miRNA-155, has also been shown to be expressed in astrocytes and been implicated in various CNS pathologies [46], including epilepsy [47–49]. Since the activation of MMP expression, especially MMP3 and MMP9, has been linked to pro-inflammatory signaling, miR-155 might modulate their expression through the regulation of inflammation. Indeed, miR-155 was previously demonstrated to be involved in the regulation of MMP3 under inflammatory conditions in synovial fibroblasts [50, 51].

The aim of this study was to investigate whether increased MMP expression under inflammatory conditions can be attenuated by inhibition of miR-155. Therefore, we investigated MMP expression in cultured human fetal astrocytes after IL-1β stimulation and transfection with the antagomiR of miR-155. Furthermore, we characterized MMP and miR-155 expression in resected brain tissue of patients with TLE as well as during epileptogenesis in a rat TLE model.

## Methods

### Cell cultures

Primary fetal astrocyte-enriched cell cultures were derived from human fetal brain tissue (14–20 weeks of gestation) obtained from medically induced abortions. All material was collected from donors from whom a written informed consent for the use of the material for research purposes was obtained by the Bloemenhove clinic. Tissue was obtained in accordance with the Declaration of Helsinki and the Amsterdam UMC Research Code provided by the Medical Ethics Committee. Tissue samples were collected in DMEM/HAM F10 (1:1) medium (Gibco/ThermoFisher Scientific, Waltham, MA, USA), supplemented with 1% penicillin/streptomycin and 10% fetal calf serum (FCS). Primary cell cultures of astrocytes were prepared as previously described [49]. The culture medium was subsequently refreshed twice a week. Cultures reached confluence after 2–3 weeks. Astrocytes were used for analyses at passages 2–5. More than 98% of the cells in primary culture, as well as in the successive 12 passages were strongly immunoreactive for the astrocytic marker glial fibrillary acid protein (GFAP) and S100β as previously reported [45].

### Transfection and stimulation of cell cultures

Cells were plated in poly-L-lysine coated plates (5 × 10^4^ cells/well in 12-well plates for RNA analysis or 2 × 10^5^ cells/well in 6-well plates for protein analysis) and were transfected with miR-146a or miR-155 mimic pre-miRNA (mirVana miRNA mimics, Applied Biosystems, Carlsbad, CA, USA), or antisense locked nucleic acid (LNA) oligonucleotides against miR-155-5p (Ribotask ApS, Odense, Denmark). Oligonucleotides were delivered to the cells using Lipofectamine 2000 transfection reagent (Life Technologies, Grand Island, NY, USA) in a final concentration of 50 nM for a total of 24 h before the stimulation of astrocytes. Astrocytic cultures were stimulated with human recombinant (r)IL-1β (10 ng/ml; Peprotech, Rocky Hill, NJ, USA) for 24 h (for RNA analysis) or for 48 h (for protein analysis) before harvesting the cells. Viability of human cell cultures was not influenced by the stimulation with IL-1β, as shown previously [52].

### Human brain tissue

The cases included in this study were obtained from the archives of the department of (Neuro)Pathology of the Amsterdam UMC, Amsterdam, The Netherlands. A total of 16 brain specimens were examined from patients undergoing surgery for drug resistant TLE. Tissue was obtained and used in accordance with the Declaration of Helsinki and the Research Code provided by the Medical Ethics Committee. All cases were reviewed independently by two neuropathologists, and the classification of hippocampal sclerosis was based on analysis of microscopic examination as described by the International League Against Epilepsy (HS type 1, *n* = 12; HS type 2, *n* = 4) [53]. Control material was obtained during autopsy of people without a history of seizures or other neurological diseases (*n* = 10). Brain tissue was fixed in 10% buffered formalin and embedded in paraffin.

### Experimental animals

Adult male Sprague-Dawley rats (Harlan Netherlands, Horst, The Netherlands) were used in this study which was approved by the University Animal Welfare committee. The rats were housed individually in a controlled environment (21 ± 1 °C; humidity 60%; lights on 08:00 AM–8:00 PM; food and water available ad libitum).

### Electrode implantation and status epilepticus induction

Rats were anesthetized with an intramuscular injection of ketamine (74 mg/kg; Alfasan, Woerden, The Netherlands) and xylazine (11 mg/kg; Bayer AG, Leverkusen, Germany), and were placed in a stereotactic frame. In order to record hippocampal EEG, a pair of insulated stainless steel electrodes (70 μm wire diameter, tips 800 μm apart) was implanted into the left dentate gyrus (DG) under electrophysiological control, as described previously [54]. A pair of stimulation electrodes was implanted in the angular bundle. Two weeks after recovery from the operation, each rat was transferred to a recording cage (40 × 40 × 80 cm) and connected to a recording and stimulation system (NeuroData Digital Stimulator, Cygnus Technology Inc., Delaware Water Gap, PA, USA) with a shielded multi-strand cable and electrical swivel (Air Precision, Le Plessis Robinson, France). A week after habituation to the new condition, rats underwent tetanic stimulation (50 Hz) of the hippocampus in the form of a succession of trains of pulses every 13 s. Each train was of 10 s duration and consisted of biphasic pulses (pulse duration 0.5 ms, maximal intensity 700 μA). Stimulation was stopped when the rats displayed sustained forelimb clonus and salivation for several minutes, which usually occurred within 1 h.

### EEG monitoring

To determine seizure frequency, continuous EEG recordings (24 h/day) were made in all rats. Hippocampal EEG signals were amplified (10×) via a field effect transistor that connected the headset to an amplifier (20×; CyberAmp, Axon Instruments, Burlingame, CA, USA), band-pass filtered (1–60 Hz) and digitized by a computer. A seizure detection program (Harmonie, Stellate Systems, Montreal, Canada) sampled the incoming signal at a frequency of 200 Hz per channel. All EEG recordings were visually screened and seizures were confirmed by trained human observers. Seizures were characterized by synchronized high-voltage amplitude oscillations and were scored when the amplitude increased more than 2-fold and lasted for at least 10 s.

### Tissue preparation

For in situ hybridization, rats were deeply anesthetized with pentobarbital (Euthasol, AST Farma, Oudenwater, The Netherlands, 60 mg/kg i.p.) and perfused via the ascending aorta (300 ml 0.37% Na_2_S/300 ml 4% paraformaldehyde in 0.1 M phosphate buffer, pH 7.4). Rats were perfused at three different time points after SE, each corresponding to the phases of epileptogenesis: the acute phase (1 day post-SE, *n* = 5), the latent phase (1 week post-SE, absence of electrographic seizures, *n* = 3), and the chronic phase (3–4 months post-SE, recurrent spontaneous electrographic seizures are evident, *n* = 6) [55]. Control rats (*n* = 4) that were implanted with EEG electrodes but not stimulated were also included. The brains were post-fixated overnight, dissected and paraffin embedded. Tissue was sectioned at 6 μm and mounted on pre-coated glass slides (Star Frost, Waldemar Knittel, Braunschweig, Germany).

For RT-qPCR analysis, rats were decapitated 1 day after SE (acute phase, *n* = 5), 1 week after SE (latent phase, *n* = 6) or 3–4 months after SE (chronic phase, *n* = 5). Control rats (*n* = 10) included young rats (*n* = 5) and age-matched controls for the chronic stage (*n* = 5). The brain was dissected and the parahippocampal cortex (PHC), which includes mainly the entorhinal cortex and parts of the perirhinal and posterior piriform cortex, was removed by incision at the ventro-caudal part underneath the rhinal fissure until approximately 5 mm posterior to bregma, as well as the hippocampus. The hippocampus was sliced into smaller parts (200–300 μm) and the DG and Cornu Ammonis (CA1) regions were cut out of the slices in 4 °C saline solution under a dissection microscope. All material was frozen on dry ice and stored at − 80 °C until use.

### RNA isolation and real-time quantitative PCR analysis

For RNA isolation, cell cultures, frozen human brain tissue, or frozen rat brain tissue were homogenized in 700 μl Qiazol Lysis Reagent (Qiagen Benelux, Venlo, The Netherlands). Total RNA, including small RNAs, was isolated using the miRNeasy Mini kit (Qiagen Benelux, Venlo, The Netherlands) according to manufacturer’s instructions. The concentration and purity of RNA were determined using a Nanodrop 2000 spectrophotometer (Thermo Fisher Scientific, Wilmington, DE, USA). To evaluate mRNA expression, 250 ng of cell culture-derived total RNA was reverse-transcribed into cDNA using oligo dT primers. The primers used for the study are listed in (Additional file 1: Table S1). The geometric mean of elongation factor 1α (EF1α) and chromosome 1 open reading frame 43 (C1orf43) expression levels was used for the normalization of RT-qPCR in human tissue and cell cultures; the geometric mean of glyceraldehyde 3-phosphate dehydrogenase (GAPDH) and TATA-box-binding protein (TBP) expression levels was used for the normalization of RT-qPCR in rat tissue. The PCR mix and cycling conditions were used as previously described [56].

The expression of miR-155-5p and U6B small nuclear RNA (RNU6B) was analyzed using TaqMan microRNA assays (Applied Biosystems, Foster City, CA, USA). cDNA was generated using TaqMan MicroRNA reverse transcription kit (Applied Biosystems, Foster City, CA, USA) according to the manufacturer’s instructions. The PCRs were run on the Roche LightCycler 480 (Roche Applied Science, Basel, Switzerland) with a 384-multiwell format.

Quantification of data was performed using LinRegPCR in which  the baseline correction and window-of-linearity are determined for each sample separately, followed by a linear regression analysis on the Log (fluorescence) per cycle to fit a straight line through the PCR data set. The slope of this line is used to determine the PCR efficiency of each individual sample. The mean PCR efficiency per amplicon and the Ct value per sample are used to calculate a starting concentration N0 per sample, which is expressed in arbitrary fluorescence units [57, 58]. The starting concentration N0 of each specific product was then divided by the geometric mean of the starting concentrations N0 of the reference genes, and this ratio was compared between groups. This value was further normalized to the corresponding control condition. The control condition used for RT-qPCR experiments in primary cultures was the condition stimulated by IL-1β due to undetectable/very low expression of MMP3 and MMP9 in untreated control.

### Western blot analysis

Cells were harvested at 48 h after treatment. The cells were washed with ice-cold PBS and homogenized in ice-cold lysis buffer (50 mM Tris-HCl pH 7.4, 150 mM of NaCl, 1% NP-40, 0.5% sodium deoxycholate) supplemented with protease inhibitor (EDTA-free protease mixture inhibitor and phosphatase inhibitor (Roche Diagnostics, Almere, the Netherlands)) by incubating on ice for 10 min and collected using a cell scraper. The homogenates were centrifuged at 12,000 x* g* for 10 min and the supernatant was used for further analysis. Protein content was determined using the bicinchoninic acid method [59]. Equal amounts of proteins (5 μg/lane for culture samples or 20 μg/lane for tissue samples) were separated using sodium dodecylsulfate-polyacrylamide gel electrophoresis (SDS-PAGE). Subsequently, separated proteins were transferred onto polyvinylidene difluoride membranes (Immobilon-P; Merck, Darmstadt, Germany) for 90 min at 100 V, using a wet electroblotting system (BioRad, Hercules, CA, USA). Blots were blocked for 1 h in 5% non-fat dry milk in Tris-buffered saline-Tween (TBS-T; 20 mM Tris, 150 mM NaCl, 0.1% Tween 20, pH 7.5). Blots were incubated overnight with primary antibodies anti-MMP3 (1:200 mouse monoclonal, clone SL-1 IIIC4, EMD Millipore, Temecula, CA, USA) or anti-β-actin (1:50,000 monoclonal mouse, clone C4, Merck, Darmstadt, Germany). After several washes in TBS-T/ 5% non-fat dry milk, blots were incubated with secondary antibodies goat anti-mouse IgG2b (for MMP3) or goat anti-mouse IgG1 coupled to horseradish peroxidase (both 1:2500; Dako, Glostrup, Denmark) for 1 h. After several washes in TBS-T, immunoreactivity was visualized using ECL PLUS Western blotting detection reagent (GE Healthcare Europe, Diegen, Belgium). Expression of β-actin was used as loading control. Chemiluminescent signal was detected using ImageQuant LAS 4000 analyzer (GE Healthcare, Eindhoven, the Netherlands). Precision Plus Protein Dual Color Standards (Bio-Rad, Richmond, CA, USA) was used to determine the molecular weight of the proteins. For the quantitative analysis of the blots and in-situ micrographs the band intensities were measured densitometrically using ImageJ software (U.S. National Institutes of Health, Bethesda, MD, USA).

### In situ hybridization on human and rat brain tissue

Paraffin-embedded brain tissue was deparaffinized in xylene and rinsed in ethanol (2 × 100%, 70%) and sterile water. Antigen retrieval was performed using a pressure cooker in sodium citrate buffer, pH 6.0, at 121 °C for 10 min. The oligonucleotide probe for miR-155 (Additional file 1) contained LNA modification, 2-o-methyl modification and digoxygenin (DIG) label (RiboTask ApS, Odense, Denmark). Sections were incubated with the probe (1:750 dilution) in hybridization mix (600 mM NaCl, 10 mM HEPES, 1 mM EDTA, 5x Denhardts, 50% Formamide) for 1 h at 56 °C. Sections were washed with saline-sodium citrate for 2 min, 0.5x for 2 min, 0.2x for 1 min (in agitation). After washing with sterile PBS, sections were blocked for 15 min with 1% BSA, 0.02% Tween 20 and 1% normal goat serum. Hybridization was detected with alkaline phosphatase (AP) labeled with anti-DIG (Roche Applied Science, Basel, Switzerland). Nitro-blue tetrazolium chloride (NBT)/5-bromo-4-chloro-3-indolyl phosphate p-toluidine salt (BCIP) was used as chromogenic substrate for AP (1:50 diluted in NTM-T buffer (100 mM Tris, pH 9.5; 100 mM NaCl; 50 mM MgCl_2_; 0.05% Tween 20)). Negative control assays were performed without probes (sections were blank).

For double-staining, the sections were first processed for in situ hybridization, followed by immunohistochemistry. Slides were washed with PBS and incubated for 1 h at room temperature with primary antibodies in PBS; mouse anti-GFAP (1:4000, Sigma-Aldrich, St. Louis, MO, USA), mouse anti-NeuN (1:2000, MAB377, Chemicon, Temecula, CA, USA), mouse anti-CD34 (1:600, Immunotech, Monrovia, CA, USA) and mouse anti-CR3/43 (1:100, Agilent, Santa Clara, CA, USA) or rabbit anti-IBA-1 (1:2000, Wako Chemicals, Neuss, Germany). After washing with PBS, sections were stained with a polymer-based peroxidase immunohistochemistry detection kit (Brightvision plus kit, ImmunoLogic, Duiven, The Netherlands) according to the manufacturer’s instructions. Signal was detected using the chromogen 3-amino-9-ethylcarbazole (Sigma-Aldrich, St. Louis, MO, USA).

### Evaluation of in situ hybridization

Expression of miR-155 was quantitatively analyzed in the DG and CA1 of human brain tissue and in the DG, CA1 and entorhinal cortex of rat brain tissue by measuring the optical density using ImageJ. Since the quantitative analysis does not discriminate between different cell types, a semi-quantitative analysis was also performed in which miR-155 expression was assessed in neurons, cells with glial morphology and blood vessels. The intensity of the staining was evaluated using a scale of 1–4 (1: no; 2: weak; 3: moderate; 4: strong staining). The score represents the predominant cell staining intensity found in each case. Additionally, the relative number of positive cells (0: no; 1: single to 10%; 2: 11–50%; 3: > 50%) was also evaluated. The in situ reactivity score (IRS) was calculated by multiplying the intensity score by the relative number score. The analysis was performed by two researchers that were blinded to group assignments.

### Statistical analysis

Statistical analyses were performed using IBM SPSS Statistics 21. Comparisons between multiple groups were done using the Kruskal-Wallis test, Mann-Whitney *U* test was used to compare two groups. A value of *p* < 0.05 was assumed to indicate significant difference.

## Results

### MMP expression in human fetal astrocytes after IL-1β stimulation

**Fig. 1:**
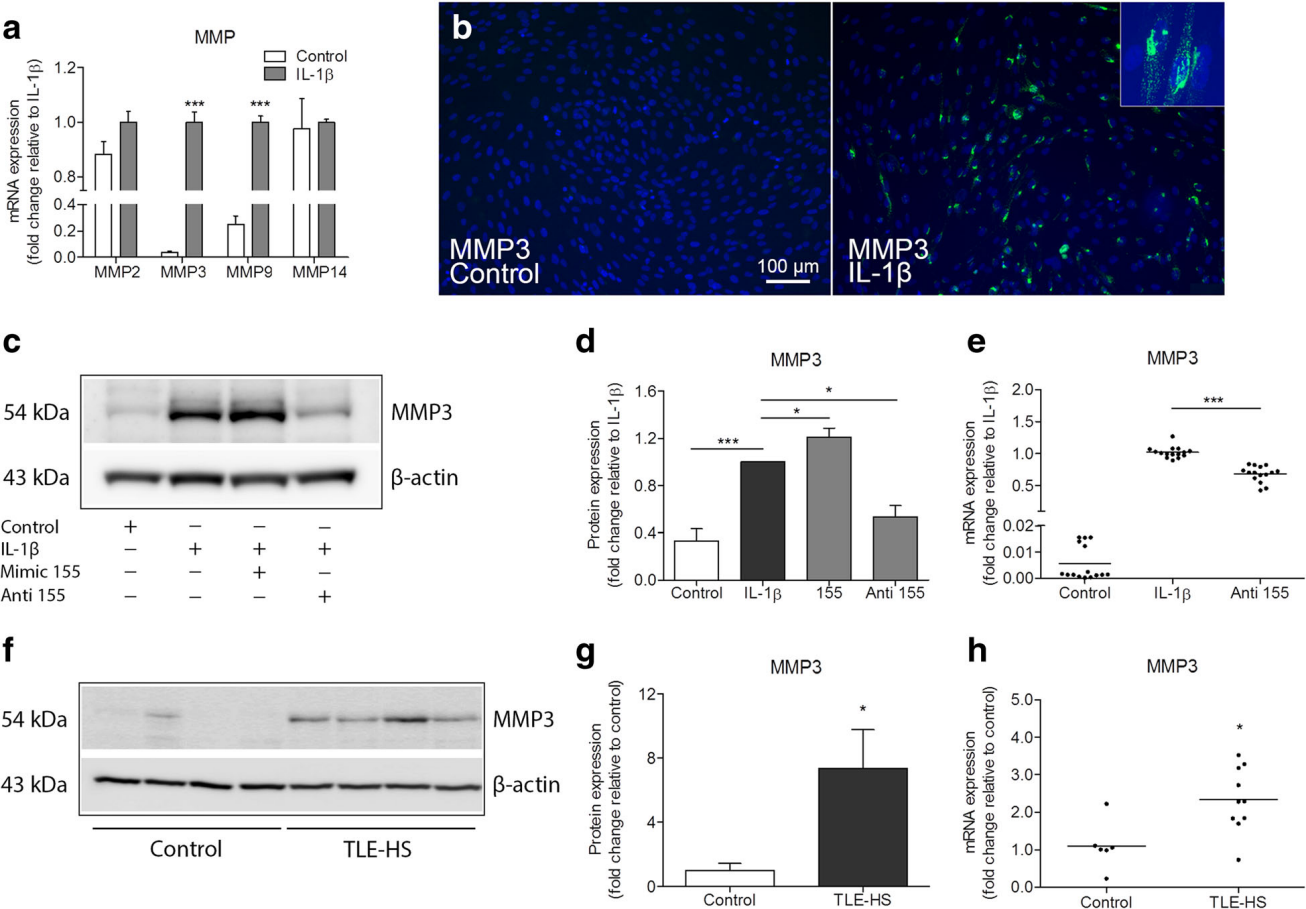
Increased MMP3 expression under pathological conditions can be modulated by miR-155 in human astrocytes. **a** RT-qPCR expression analysis demonstrated increased expression of MMP3 (*p* < 0.001) and MMP9 (*p* < 0.001), but not MMP2 and MMP14 mRNA following IL-1β stimulation in human fetal astrocytes. **b** Immunocytochemistry revealed MMP3 immunoreactive cells 24 h after the stimulation of primary human fetal astrocytes with IL-1β (10 ng/ml), while the immunoreactivity in control cells was not observed; the inset demonstrate a magnified image of cells expressing MMP3 after IL-1β stimulation. **c** Western blot for MMP3 in human fetal astrocytes. **d** A semi-quantitative analysis of MMP3 immunoreactivity showed an upregulation of MMP3 after IL-1β stimulation (*p* < 0.001, *n* = 5); the cells transfected with miR-155 mimic prior to the stimulation had a further upregulation of MMP3 (*p* < 0.05, *n* = 5), and the cells transfected with miR-155 antagomiR showed a decreased expression of MMP3 (*p* < 0.05, *n* = 5) compared to non-transfected cells. **e** RT-qPCR showed decreased MMP3 mRNA expression in the cells transfected with the antagomiR of miR-155 after IL-1β stimulation (*p* < 0.001, *n* = 5). **f** Western blot for MMP3 in TLE-HS specimens compared to autoptic control specimens. **g** A semi-quantitative analysis of MMP3 immunoreactivity showed an upregulation of MMP3 (*p* < 0.05) in TLE-HS (*n* = 6) compared to autoptic control (*n* = 5). **h** RT-qPCR analysis showed that MMP3 mRNA was increased (*p* < 0.05) in a subset of patients with TLE-HS (*n* = 10) compared to autoptic control (*n* = 6). Gene expression was represented as normalized to the geometric mean of the expression of two housekeeping genes. Anti = antagomiR; *p < 0.05, **p < 0.01, Mann-Whitney *U* test, error bars depict the standard error of the mean

Constitutively, MMP2 and MMP14 were expressed highly in primary cultures of human fetal astrocytes, whereas for MMP3 and MMP9 a low expression was observed (Additional file 2). RT-qPCR analysis demonstrated that gene expression of MMP3 and MMP9 was increased following IL-1β stimulation (*p* < 0.001, Fig. 1a), and the induction was especially prominent for MMP3. Gene expression of MMP2 and MMP14 did not change. Immunocytochemistry (Fig. 1b) and Western blot analysis (*p* < 0.001, Fig. 1c, d) showed that MMP3 protein was also increased following IL-1β stimulation.

### Modulation of MMP3 expression in human fetal astrocytes by miRNA-155 after IL-1β stimulation

Since MMP3 gene and protein expression was increased in human fetal astrocytes after IL-1β stimulation, we investigated whether MMP3 expression could be modulated. Therefore, human fetal astrocytes were transfected with miR-146a mimic, miR-155 mimic or miR-155 antagomiR and treated with IL-1β. TaqMan RT-qPCR analysis confirmed that miR-155 was increased after IL-1β stimulation (*p* < 0.001; Additional file 3), while transfection with miR-155 antagomiR did not alter miRNA levels, as previously shown [49]. Western blot analysis showed that inhibition of miR-155 by antagomiR resulted in a decreased MMP3 expression (*p* < 0.05) when compared to IL-1β-stimulated condition (Fig. 1c, d), while miR-146a did not reduce MMP3 protein expression (data not shown). In contrast, overexpression of miR-155 led to a further increase (*p* < 0.05) of MMP3 protein expression after IL-1β stimulation (Fig. 1c, d). RT-qPCR analysis showed that MMP3 mRNA was also decreased in astrocytes transfected with miR-155 antagomiR (*p* < 0.001) as compared to non-transfected cells under IL-1β stimulation (Fig. 1e; Additional file 4).

### MMP3 expression in the hippocampus of TLE-HS patients

Following the findings in human astrocytic cell cultures, we investigated the expression of MMP3 in the hippocampal specimens resected from TLE-HS patients. The clinical features of the human subjects included in this study are summarized in Table 1. Western blot analysis showed a weak MMP3 immunoreactivity in autopsy control samples, while MMP3 expression was more evident in TLE-HS specimens (Fig. 1f, g). Similarly, RT-qPCR analysis showed an increased MMP3 mRNA expression (*p* < 0.05) in the TLE-HS group as compared to controls (Fig. 1h).

**Table 1:** Clinical findings of human samples used for RT-qPCR and in situ hybridization

Experiment	Pathology	*n*	Age	Gender m/f	HS ILAE* Type 1/type 2	Duration of epilepsy (years)	Age at onset	Number of seizures (per month)
RT-qPCR	Control	6	63 (25–86)	4/2	–	–	–	–
	TLE-HS	16	35 (24–49)	9/7	12/4	20 (5–41)	15 (3–34)	19 (1–36)
ISH	Control	5	48 (31–64)	3/2	–	–	–	–
	TLE-HS	10	35 (24–49)	7/3	7/3	20 (5–41)	16 (3–34)	13 (2–32)

*TLE* temporal lobe epilepsy, *HS* hippocampal sclerosis, *ISH* in situ hybridization, *m* male, *f* female. Values are given as mean (minimum–maximum)

*According to Blümcke et al. 2013 [53]

### miR-155 expression in the hippocampus of TLE-HS patients

**Fig. 2:**
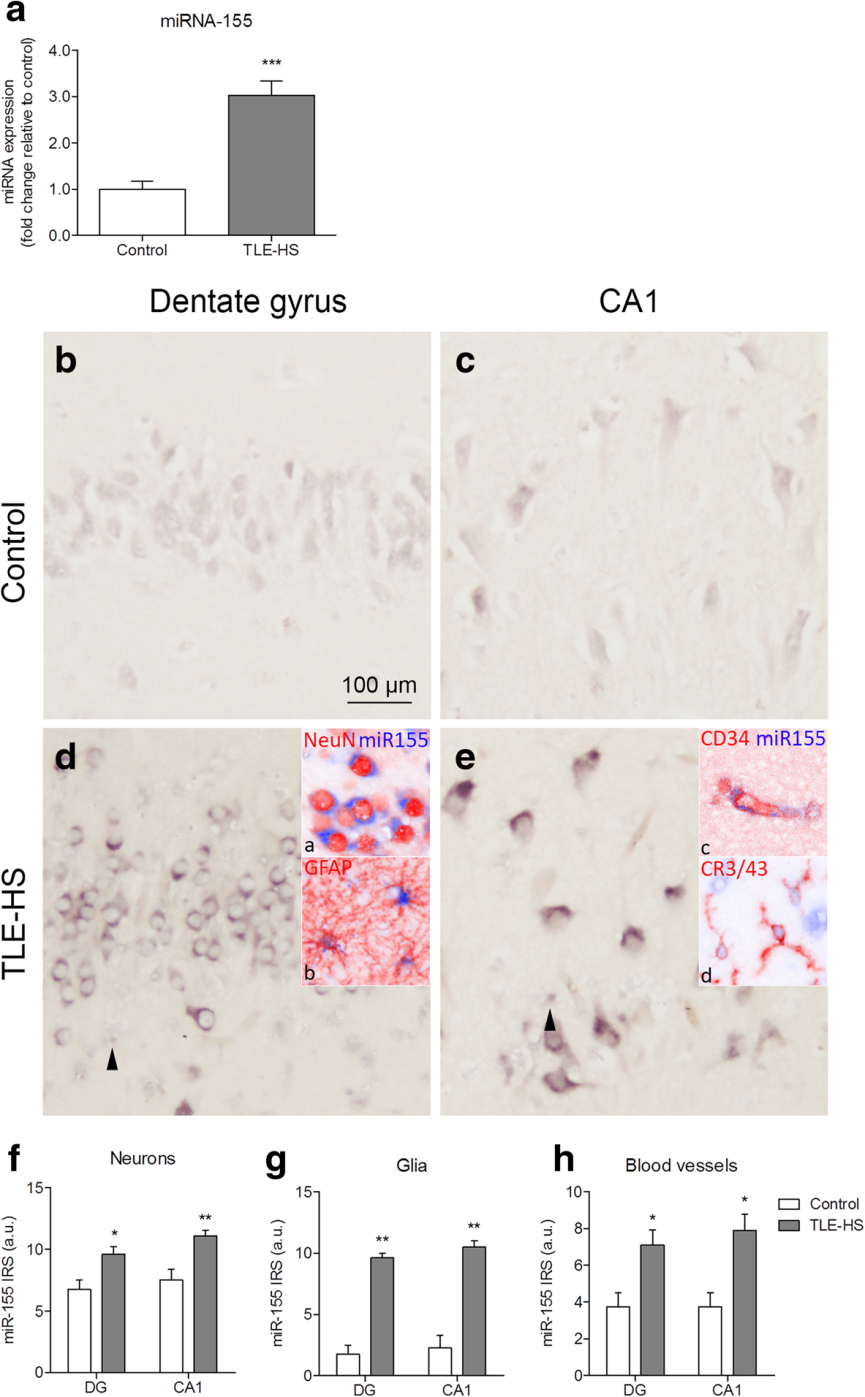
miR-155 expression in TLE-HS. **a** miR-155 expression was increased in TLE-HS patients relative to controls evaluated by TaqMan RT-qPCR (*p* < 0.001). **b**–**e** In situ hybridization for miR-155 in the human hippocampus. A moderate neuronal miR-155 expression was observed in the control hippocampus (**b**, **c**). **d**, **e** miR-155 expression was increased in both neurons and in cells with glial morphology compared to control; co-localization was found with neurons (NeuN; **d**, inset a), astrocytes (GFAP; **d**, inset b), endothelial cells (CD34; **e**, inset c) and microglia (CR3/43; **e**, inset d). Arrowheads indicate cells with glial morphology. **f–h** A semi-quantitative analysis of miR-155 IRS in neurons, cells with glial morphology, and blood vessels. Scale bar 100 μm; *p < 0.05, **p < 0.01, ***p < 0.001, Mann-Whitney *U* test, error bars depict standard error of the mean

We investigated the expression of miR-155 in the human hippocampus. TaqMan RT-qPCR analysis revealed an increased expression of miR-155 (*p* < 0.001) in the TLE-HS compared to controls (Fig. 2a). The cellular distribution of miR-155 was investigated by in situ hybridization in the DG and CA1. In autopsy control tissue, a moderate expression of miR-155 was observed in neurons, while a weak expression was found in cells with glial morphology (Fig. 2b, c). In TLE-HS, a moderate to strong miR-155 expression was observed in both neurons and cells with glial morphology (Fig. 2d, e). In order to confirm the observations of cell morphology, we performed a double immuno-labelling of miR-155 with the markers for different cell types. miR-155 was co-localized with the markers for neurons (NeuN), astrocytes (GFAP), microglia (CR3/43), and endothelial cells (CD34) in TLE-HS tissue (Fig. 2d, insets a, b; Fig. 2e, insets c, d). Quantitative analysis (Additional file 5) of miR-155 expression showed a trend toward increased miR-155 expression in the DG in TLE-HS tissue as compared to control. Since this analysis does not discriminate between different cell types, we also performed a semi-quantitative analysis (see the “Methods” section for description) which showed that the miR-155 IRS was increased for neurons (DG *p* < 0.05, CA1 *p* < 0.01), cells with glial morphology (DG, CA1 *p* < 0.01), as well as for blood vessels (DG, CA1 *p* < 0.05) compared to controls (Fig. 2f, g, h; Table 2).

**Table 2:** In situ reactivity scores of miR-155 in the human hippocampus

	DG			CA1		
	Neurons	Glia	Vessels	Neurons	Glia	Vessels
Control	6 (6–9)	1 (1–4)	3 (3–6)	7.5 (6–9)	2.5 (0–4)	3 (3–6)
TLE-HS	9 (6–12)*	9 (9–12)**	6 (4–12)*	12 (9–12)**	10.5 (9–12)**	9 (4–12)*

miR-155 in situ reactivity scores are given as median (minimum–maximum). This score was defined as intensity score (*1* no; *2* weak; *3* moderate; *4* strong immunoreactivity) multiplied by relative number score (*0* no; *1* single to 10%; *2* 11–50%; *3* > 50%); *p < 0.05, **p < 0.01

### MMP3 expression during epileptogenesis in a rat TLE model

**Fig. 3:**
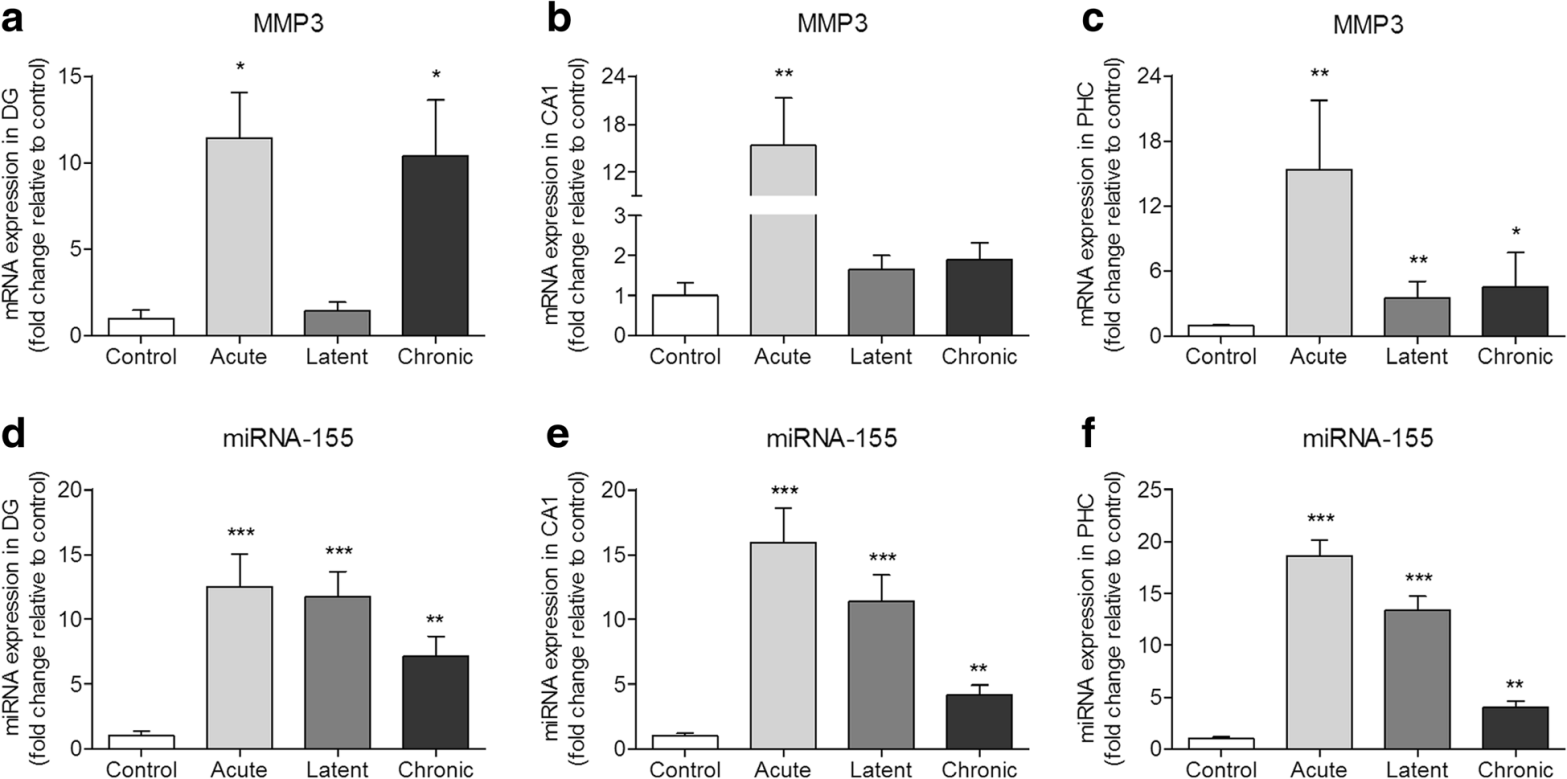
MMP3 and miR-155 expression in the rat TLE model. **a–c** RT-qPCR analysis of MMP3 expression in the rat hippocampus. **a** MMP3 was increased in the DG at the acute (1 day post-SE, *p* < 0.05) and chronic (3–4 months post-SE, *p* < 0.05) stages. **b** MMP3 was increased in the CA1 at the acute stage (*p* < 0.01). **c** MMP3 was increased in the PHC at the acute (*p* < 0.01), latent (1 week post-SE, *p* < 0.01), and chronic (*p* < 0.05) stages; **d**–**f** TaqMan RT-qPCR analysis of miR-155 expression in the DG (**d**), CA1 (**e**), and PHC (**f**) regions of the rat hippocampus. *p < 0.05, **p < 0.01, ***p < 0.001, Mann-Whitney *U* test, error bars depict standard error of the mean

We further investigated MMP3 expression in the brain of rats after SE. RT-qPCR analysis showed that MMP3 was increased in the DG 1 day and 3–4 months after SE induction (respectively, acute and chronic stages; *p* < 0.05; Fig. 3a); increased expression was also seen during the acute stage (*p* < 0.01) in the CA1 region (Fig. 3b) and during all 3 stages of epileptogenesis (acute *p* < 0.01; latent *p* < 0.01; chronic *p* < 0.05) in the PHC (Fig. 3c) of the rats*.*

### miR-155 expression during epileptogenesis in a rat TLE model

We investigated the expression of miR-155 at different time points during epileptogenesis in the rat brain after SE. miR-155 expression was increased in the DG, CA1, and PHC during the acute (*p* < 0.001), latent (*p* < 0.001), and chronic (*p* < 0.01) phases as compared to control (Fig. 3d–f). miR-155 expression peaked at the acute phase in all three regions, but was also increased during the latent and chronic phases as compared to control.

**Fig. 4:**
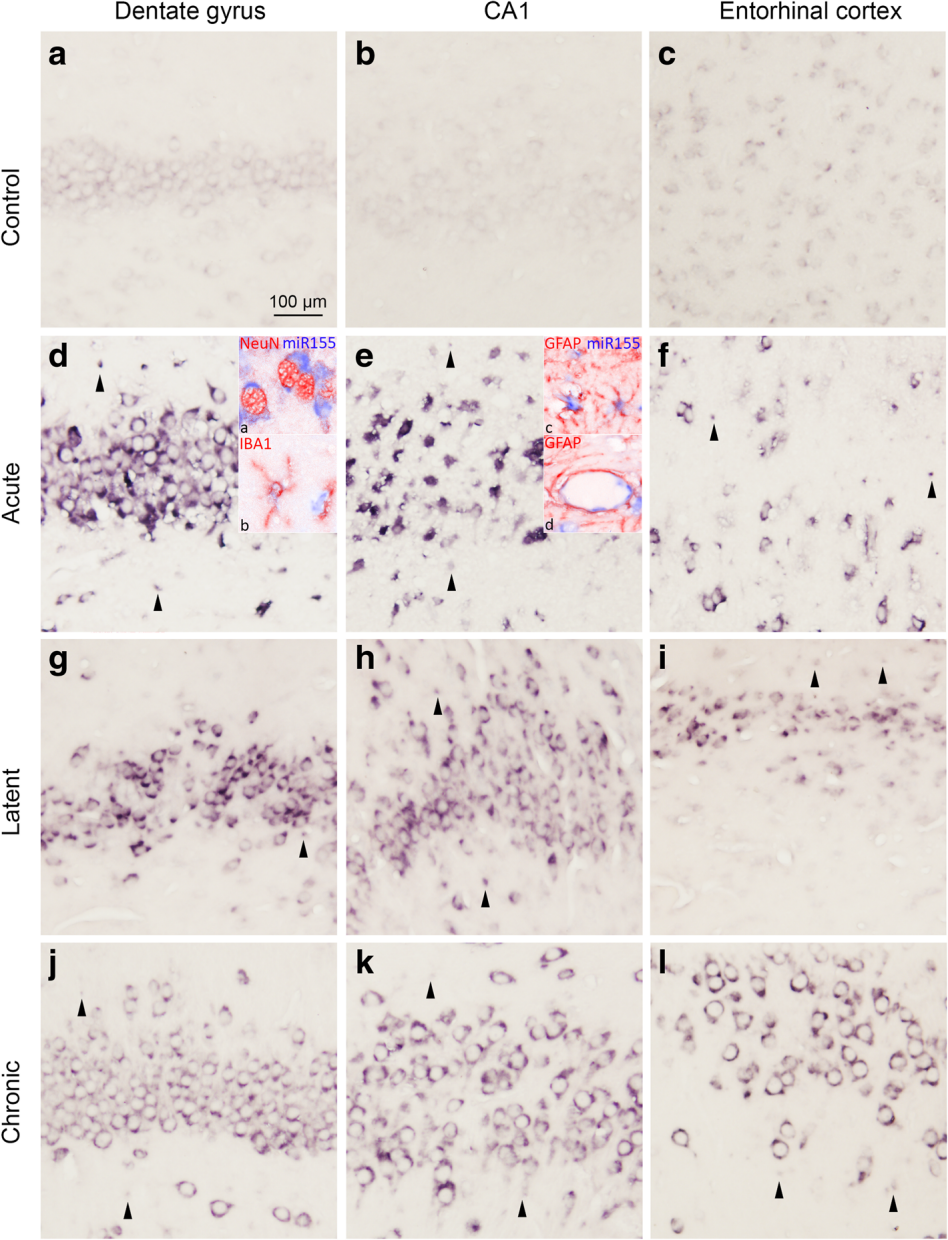
In situ hybridization of miR-155 in the rat TLE model. **a–c** A weak miR-155 expression was seen in neurons of the control rats in the DG, CA1, and entorhinal cortex (EC). d–l Increased expression compared to control was observed in both neurons and glia of the rats at the acute, latent, and chronic stages. **d** Co-localization was found with neurons (NeuN; **d**, inset a), microglia (IBA-1; **d**, inset b), and astrocytes (GFAP; **e**, inset c). Additionally, miR-155 expression was seen in blood vessels (**e**, inset d). Scale bar 100 μm; arrowheads indicate cells with glial morphology

**Fig. 5:**
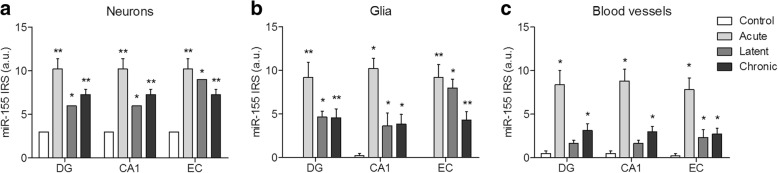
miR-155 expression in the rat TLE model. A semi-quantitative analysis of miR-155 expression in neurons (**a**), glia (**b**), and blood vessels (**c**) showed that miR-155 was upregulated in these cell types during all phases of epileptogenesis in the dentate gyrus (DG), CA1, and entorhinal cortex (EC). * indicates the comparison to controls for each brain region. *p < 0.05, **p < 0.01, ***p < 0.001, Mann-Whitney *U* test, error bars depict standard error of the mean

The cellular distribution of miR-155 was investigated by in situ hybridization. A weak expression of miR-155 was observed in neurons in control rat hippocampus (Fig. 4a–c). Strong miR-155 expression was observed in the DG, CA1, and entorhinal cortex (EC) already at the acute phase following SE (Fig. 4d–f) and was also moderate to strong throughout the course of epileptogenesis at the latent (Fig. 4g–i) and chronic phases (Fig. 4j–l). Double labelling showed that miR-155 was expressed in neurons, astrocytes, and microglia (Fig. 4d insets a, b; Fig. 4e, inset c). In addition, miR-155 immunoreactivity was visible in blood vessels (Fig. 4e, inset d). Quantitative (Additional file 6) as well as semi-quantitative analysis confirmed an increased expression of miR-155 in neurons, cells with glial morphology, and blood vessels in the hippocampus during the acute, latent, and chronic phases (Fig. 5a–c; Table 3).

**Table 3:** In situ reactivity scores of miR-155 in the rat hippocampus and entorhinal cortex

	DG			CA1			EC		
	Neurons	Glia	Vessels	Neurons	Glia	Vessels	Neurons	Glia	Vessels
Control	3	0	0.5 (0–1)	3	0 (0–1)	0.5 (0–1)	3	0	0 (0–1)
Acute	12 (6–12)**	12 (4–12)**	8 (4–12)*	12 (6–12)**	12 (6–12)*	8 (6–12)*	12 (6–12)**	9 (4–12)**	8 (4–12)*
Latent	6*	4 (4–6)*	2 (1–2)	6*	4 (1–6)*	2 (1–2)	9*	9 (6–9)*	2 (1–4)*
Chronic	6 (6–9)**	4 (1–9)**	2 (1–6)*	6 (6–9)**	4 (1–9)*	3 (1–6)*	6 (6–9)**	4 (1–9)**	2 (1–6)*

miR-155 in situ reactivity scores are given as median (minimum–maximum). This score is defined as intensity score (*1* no; *2* weak; *3* moderate; *4* strong immunoreactivity) multiplied by relative number score (*0* no; *1* single to 10%; *2* 11–50%; *3* > 50%); *DG*, dentate gyrus; *EC*, entorhinal cortex; *p < 0.05, **p < 0.01

## Discussion

We demonstrated that MMP3 expression was increased in primary cultures of human fetal astrocytes following stimulation of the cells with the pro-inflammatory cytokine IL-1β. Inhibition of miR-155 attenuated the increased expression of MMP3 after the IL-1β stimulation. Increased expression of both MMP3 and miR-155 was also evident in the hippocampus of TLE-HS patients and throughout epileptogenesis in rats. These results are further discussed in detail in the following paragraphs.

### miR-155 modulates MMP3 in human astrocytes

MMPs are key proteases participating in the remodeling of the ECM. We reported previously that the expression of MMP2, MMP3, MMP9, and MMP14 was increased and dynamically regulated in the brain of rats at different stages of post-SE epileptogenesis [26]. We have investigated the expression of these genes further in a primary culture of human astrocytes. Various stimuli, such as cytokines, growth factors, reactive oxygen species, or cell–ECM interactions are known to be responsible for the transcriptional activation of MMPs [33, 60–62]. In our experiments, the stimulation of human astrocytes with the pro-inflammatory cytokine IL-1β resulted in increased expression of MMP3. We further demonstrated that the increased expression of MMP3 after IL-1β stimulation was attenuated upon inhibition of miR-155 expression in the astrocytes by antagomiR transfection. To our knowledge, MMP3 has not been predicted or shown to be a direct target of miR-155, suggesting an indirect mechanism of miR-155-mediated inhibition of MMP3. The upregulation of MMP3 following pro-inflammatory stimuli has been previously reported, and it was proposed to be dependent on the activation of activator protein 1 (AP-1) and nuclear factor kappa-light-chain-enhancer of activated B cells (NF-κB) transcription factors [32, 63–65]. These transcription factors have been associated with apoptosis, innate immune response, and inflammation [66, 67]. miR-155 production has been shown to be involved in the feedback regulation of these pathways [68, 69] and been attributed a bidirectional role in the regulation of pro-inflammatory signaling [43, 70, 71]. Therefore, it is likely that the inhibition of miR-155 exerts its effect on MMP3 expression via interference with the members of pro-inflammatory signaling pathways.

### Increased expression of MMP3 in TLE-HS and during epileptogenesis in a rat TLE model

We found that MMP3 expression was increased in the hippocampus of TLE-HS patients. In healthy CNS, the expression of MMP3 is low, but it can be increased in pathology, such as ischemia, trauma, or neurodegenerative disorders [72–75]. TLE-HS is characterized by extensive neurodegeneration in the hippocampus, chronic neuroinflammation, reactive gliosis, remodeling of the ECM, and dysfunction of the BBB [54, 76–80]. Reactive astrocytes and microglia provide a major source of pro-inflammatory molecules, including IL-1β [81–85], and as discussed in the previous paragraph, astrocytes can produce increased amounts of MMP3 upon exposure to IL-1β. Thus, the upregulation of MMP3 may be due to excessive production and release of pro-inflammatory cytokines and chronic neuroinflammation in TLE-HS.

Human TLE-HS tissue represents the end-stage of the disease and does not allow to track changes along the course of epileptogenesis. To further characterize the spatio-temporal changes of MMP3 expression, we used a rat model of post-SE epileptogenesis, which recapitulates the pathological features of TLE-HS [55]. The expression of MMP3 in the rat hippocampus was the highest during the acute phase of epileptogenesis, indicating the involvement of this protease shortly after an epileptogenic insult. Moreover, increased MMP3 expression was also observed in the DG and PHC during the chronic stage, which supports the observations made in human chronic TLE-HS specimens. MMP3 has been associated with neuroinflammation via its ability to activate microglial cells (causing them to produce superoxide, TNF-a, and IL-1β) and participation in BBB breakdown (through the proteolysis of laminin, fibronectin, and type IV collagen) [33], which can contribute to epileptogenesis.

### Increased expression of miR-155 in TLE-HS and during post-SE epileptogenesis in rats

We found increased expression of miR-155 in the brain of patients with TLE-HS and in the rat TLE model. This supports previous studies, implicating miR-155 in epilepsy [48, 86]. We have extended these data by characterizing the cellular distribution of miR-155 in both human and rat brain. While miR-155 expression was low in control rats, it was elevated in different sub-regions of the hippocampus and in the PHC throughout epileptogenesis. miR-155 expression was most evident in principal neuronal layers of the hippocampus as well as in glial cells, including astrocytes and microglia. miR-155 has been previously suggested to play a role in modulation of the inflammatory activation in microglia [87–89] and was shown to be upregulated in astrocytes and microglia in response to pro-inflammatory stimuli, such as IL-1β, lipopolysaccharide (LPS), interferon gamma (IFN-γ), and tumor necrosis factor alpha (TNF-α) [48, 49]. We also observed increased expression of miR-155 in blood vessels in both human and rat hippocampus, implying a role of miR-155 in the BBB dysfunction that occurs in epilepsy. miR-155 was previously found to contribute to the BBB permeability [90]. Moreover, MMP3 knockout mice were demonstrated to have less degradation of tight junction proteins and the basal lamina upon acute pro-inflammatory challenge [91]. Thus, miR-155 was expressed in various cell types in the hippocampus and, similar to MMP3, was increased shortly after an epileptogenic stimulus and elevated throughout epileptogenesis.

### miR-155 inhibition as a therapeutic anti-epileptogenic strategy

The increased expression of MMPs may contribute to the epileptogenic process by contributing to the BBB dysfunction and exacerbating brain inflammation. Furthermore, MMPs have been proposed as candidate proteases involved in the degradation of the perineuronal nets (PNNs) in epilepsy, and the treatment of rats with an MMP inhibitor doxycycline was demonstrated to prevent PNN degradation in a rat kindling model [92]. MMP3 protein localization has been observed in the perineuronal space [93], and MMP3 substrates include various proteoglycans constituting PNNs, such as aggrecan, neurocan, brevican, versican, phosphacan, and their binding partners, tenascin-R, hyaluronan, and link proteins [94]. This suggests that the inhibition of excessive MMP3 production shortly after a brain insult may alleviate epileptogenic processes such as neurodegeneration and BBB disruption. Indeed, inhibition of MMPs with broad-spectrum MMP inhibitors protected PNNs and had an anti-epileptogenic effect [92]. However, the existing MMP inhibitors have been reported to lack specificity and cause serious side effects [95]. Our data show that inhibition of miR-155 attenuates MMP3 expression under inflammatory conditions in vitro, and therefore, we propose that miR-155 could be a potential therapeutic target, which needs to be further investigated in vivo.

The modulation by inflammation-associated miRNAs has been proposed as a therapeutic approach to treat epilepsy and reduce brain damage. It has been reported that in vivo miR-155 inhibition supports the integrity of endothelial tight junctions, reduces brain tissue damage, improves the functional recovery in animal models after experimental ischemic stroke [96], and reduces seizure frequency in the pilocarpine TLE model [86]. Recently, we have demonstrated that intraventricular administration of miR-146a mimic resulted in a decreased seizure frequency and severity in mice [97] and a similar strategy might be used to study potential disease-modifying effects of miR-155 inhibition. The pharmacological inhibition of miR-155 may have a dual benefit: via the reduction of inflammation and by protecting the ECM through the reduction of MMP3 expression.

## Conclusions

Our experiments showed that MMP3 is dynamically regulated by seizures as shown by increased expression in TLE tissue and during different phases of epileptogenesis in the rat TLE model. MMP3 can be induced by the pro-inflammatory cytokine IL-1β and is regulated by miR-155, suggesting a possible strategy to prevent epilepsy via reduction of inflammation.

## Additional files

Additional file 1: **Table S1.** The list of oligonucleotides. (DOCX 18 kb)

Additional file 2: **Figure S1.** MMP expression levels in astrocytic culture. (PDF 93 kb)

Additional file 3: **Figure S2.** TaqMan qPCR analysis of miR-155 expression in astrocytic culture after IL-1β stimulation. (PDF 89 kb)

Additional file 4: **Figure S3.** MMP expression in astrocytic culture after transfection with antagomiR of miR-155 and miR-146a following IL-1β stimulation. (PDF 100 kb)

Additional file 5: **Figure S4.** Densitometrical quantification of miR-155 expression in TLE-HS. (PDF 84 kb)

Additional file 6: **Figure S5.** Densitometrical quantification of miR-155 expression in the rat TLE model. (PDF 89 kb)

## Abbreviations

AP-1: Activator protein 1
BBB: Blood–brain barrier
C1orf43: Chromosome 1 open reading frame 43
CA1: Cornu Ammonis 1
DG: Dentate gyrus
EC: Entorhinal cortex
ECM: Extracellular matrix
EF1α: Elongation factor 1α
GAPDH: Glyceraldehyde 3-phosphate dehydrogenase
GFAP: Glial fibrillary acidic protein
IFN-γ: Interferon gamma
IL-1β: Interleukin-1β
IRS: In situ reactivity score
LNA: Locked nucleic acid
LPS: Lipopolysaccharide
miRNA: MicroRNA
MMP: Matrix metalloproteinase
NF-κB: Nuclear factor kappa-light-chain-enhancer of activated B cells
PHC: Parahippocampal cortex
PNN: Perineuronal net
RNU6B: U6B small nuclear RNA
SE: Status epilepticus
TBP: TATA-box-binding protein
TLE-HS: Temporal lobe epilepsy with hippocampal sclerosis
TNF-α: Tumor necrosis factor alpha
