# Supplementary material for: Increased expression of matrix metalloproteinase 3 can be attenuated by inhibition of microRNA-155 in cultured human astrocytes
Source: J Neuroinflammation. 2018 Jul 21;15:211. doi: 10.1186/s12974-018-1245-y (PMC6054845; doi:10.1186/s12974-018-1245-y)
Supplement: Supplementary file 1 — Table S1. The list of oligonucleotides. (DOCX 18 kb) [file 12974_2018_1245_MOESM1_ESM.docx]

**Supplementary Table S1**

| **Human primers for RT-qPCR** | |
| --- | --- |
| MMP2 | forward: ATAACCTGGATGCCGTCGT |
|  | reverse: AGGCACCCTTGAAGAAGTAGC |
| MMP3 | forward: CTCCAACCGTGAGGAAAATC |
|  | reverse: CATGGAATTTCTCTTCTCATCAAA |
| MMP9 | forward: GCCCTTCTACGGCCACTACT |
|  | reverse: CGTCGAAGATGTTCACGTTG |
| MMP14 | forward: GCCTTGGACTGTCAGGAATG |
|  | reverse: AGGGGTCACTGGAATGCTC |
| EF1α | forward: ATCCACCTTTGGGTCGCTTT |
|  | reverse: CCGCAACTGTCTGTCTCATATCAC |
| C1orf43 | forward: GATTTCCCTGGGTTTCCAGT |
|  | reverse: ATTCGACTCTCCAGGGTTCA |
| **Rat primers for RT-qPCR** | |
| MMP3 | forward: TTGTCCTTCGATGCAGTCAG |
|  | reverse: GGGGTCCTGAGAGATTTTCG |
| GAPDH | forward: ATGACTCTACCCACGGCAAG |
|  | reverse: TACTCAGCACCAGCATCACC |
| TBP | forward: CAGGAGCCAAGAGTGAAGAAC |
|  | reverse: AGGAAATAACTCTGGCTCATAACTACT |
| **Probes for *in situ* hybridization** | |
| miR-155-5p | 5’DIG-*AmCmC*CmCmU*AmUmC*AmCmG*AmUmU*AmGmC*AmUmUmA*A-DIG3' |

**Supplementary Table S1.** The list of oligonucleotides

Human primers were designed for *Homo sapiens* and rat primers were designed for *Rattus norvegicus*; MMP – matrix metalloproteinase, EF1α - elongation factor 1α, C1orf43 - chromosome 1 open reading frame 43, GAPDH - glyceraldehyde 3-phosphate dehydrogenase, TBP - TATA-box-binding protein; miR-155-5p oligonucleotide probe for *in situ* hybridization had the following modifications: * - locked nucleic acid (LNA) modification; m - 2-o-methyl modification; DIG - digoxygenin label
