# Supplementary material for: Increased expression of matrix metalloproteinase 3 can be attenuated by inhibition of microRNA-155 in cultured human astrocytes
Source: J Neuroinflammation. 2018 Jul 21;15:211. doi: 10.1186/s12974-018-1245-y (PMC6054845; doi:10.1186/s12974-018-1245-y)
Supplement: Supplementary file 2 — Figure S1. MMP expression levels in astrocytic culture. (PDF 93 kb) [file 12974_2018_1245_MOESM2_ESM.pdf]

Supplementary Figure 1

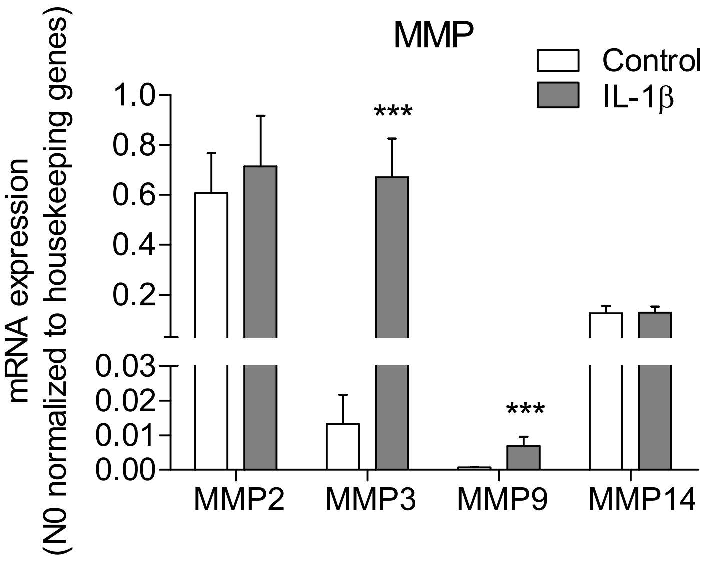

Supplementary Fig. 1. RT-qPCR analysis demonstrated increased expression of MMP3 ($p<0.001$) and MMP9 ($p<0.001$), but not MMP2 and MMP14 after IL-1β stimulation. MMP2 and MMP14 were constitutively expressed in astrocytes and their expression did not change after IL-1β stimulation, whereas MMP3 expression was induced from low expression to a level comparable with that of MMP2. MMP9 was induced, but was not highly expressed relative to the other MMPs. The data represented as normalized N0 value, which is inversely proportional to Ct value and represents estimated starting amount of mRNA in the sample (see Materials and Methods for more information).
