# Supplementary material for: Increased expression of matrix metalloproteinase 3 can be attenuated by inhibition of microRNA-155 in cultured human astrocytes
Source: J Neuroinflammation. 2018 Jul 21;15:211. doi: 10.1186/s12974-018-1245-y (PMC6054845; doi:10.1186/s12974-018-1245-y)
Supplement: Supplementary file 3 — Figure S2. TaqMan qPCR analysis of miR-155 expression in astrocytic culture after IL-1β stimulation. (PDF 89 kb) [file 12974_2018_1245_MOESM3_ESM.pdf]

Supplementary Figure 2

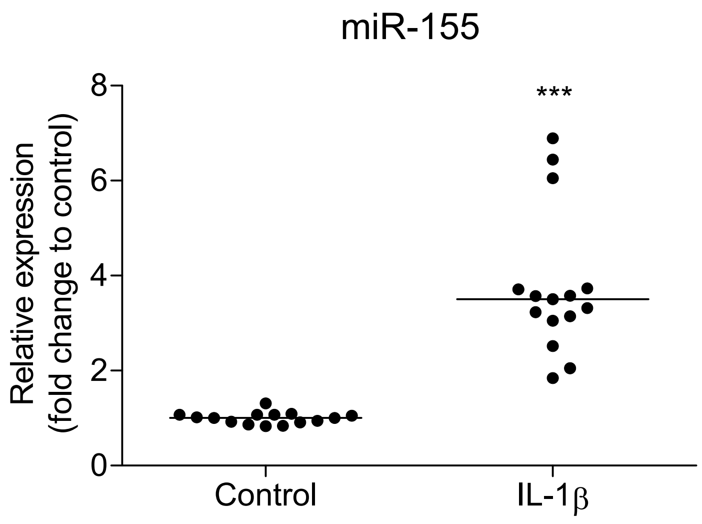

Supplementary Fig. 2. Taqman RT-qPCR analysis showed that 24 h after IL-1β stimulation miR-155 expression in primary human fetal astrocytes was increased relative to control ($p<0.001$); *** $p<0.001$, Mann-Whitney U test, error bars depict standard error of the mean.
