# Supplementary material for: Increased expression of matrix metalloproteinase 3 can be attenuated by inhibition of microRNA-155 in cultured human astrocytes
Source: J Neuroinflammation. 2018 Jul 21;15:211. doi: 10.1186/s12974-018-1245-y (PMC6054845; doi:10.1186/s12974-018-1245-y)
Supplement: Supplementary file 4 — Figure S3. MMP expression in astrocytic culture after transfection with antagomiR of miR-155 and miR-146a following IL-1β stimulation. (PDF 100 kb) [file 12974_2018_1245_MOESM4_ESM.pdf]

Supplementary Figure 3

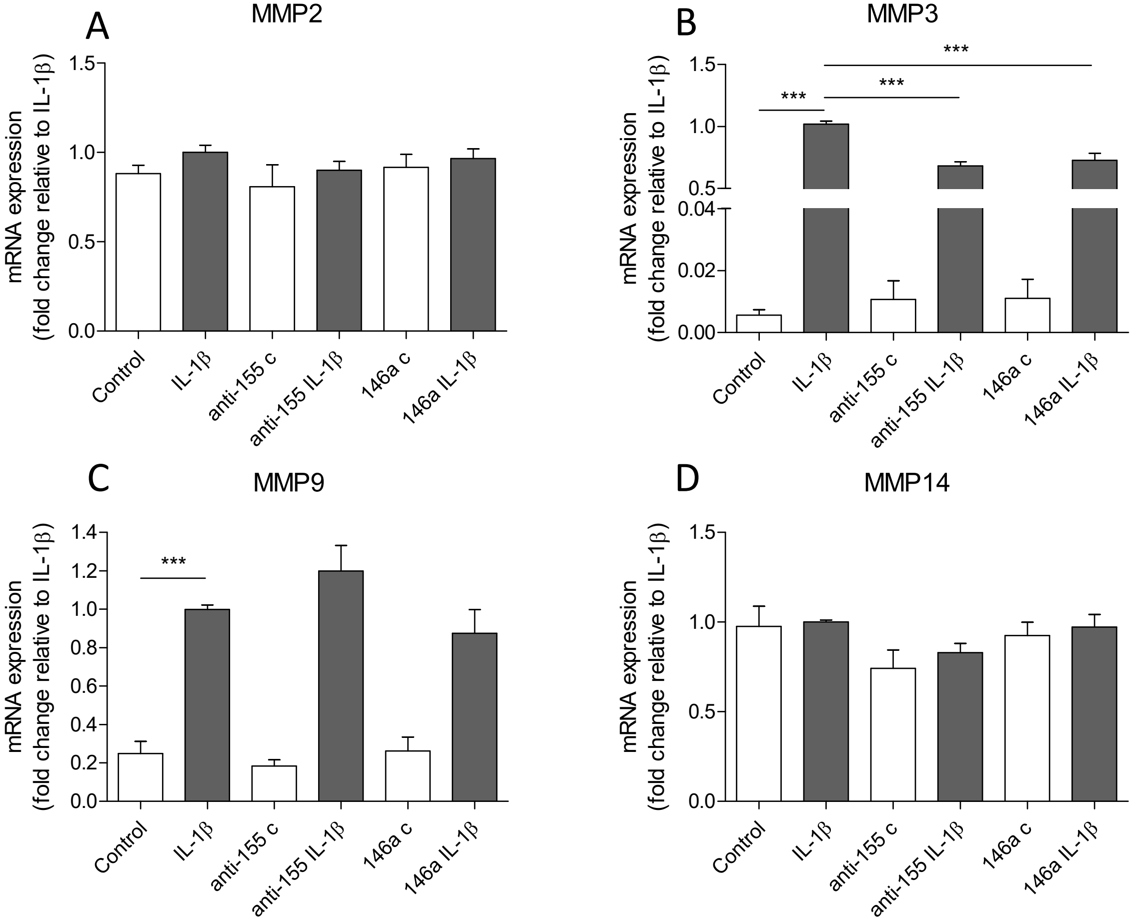

Supplementary Fig. 3. RT-qPCR analysis demonstrated an increased expression of MMP3 (**B**, p<0.001) and MMP9 (**C**, p<0.001), but not MMP2 (**A**) and MMP14 (**D**) mRNA following IL-1β stimulation in human fetal astrocytes; **(B)** MMP3 expression was decreased in cells transfected with the antagomiR of miR-155 (p<0.001) or mimic of miR-146a (p<0.001) after IL-1β stimulation; (**A-D**) The expression of MMPs did not change in transfected cells compared to control (lipofectamine vehicle) when IL-1β was not applied; *** p<0.01, Mann-Whitney U test, error bars depict the standard error of the mean; c – control, anti-155 - antagomiR of miR-155.
