# Supplementary material for: Increased expression of matrix metalloproteinase 3 can be attenuated by inhibition of microRNA-155 in cultured human astrocytes
Source: J Neuroinflammation. 2018 Jul 21;15:211. doi: 10.1186/s12974-018-1245-y (PMC6054845; doi:10.1186/s12974-018-1245-y)
Supplement: Supplementary file 5 — Figure S4. Densitometrical quantification of miR-155 expression in TLE-HS. (PDF 84 kb) [file 12974_2018_1245_MOESM5_ESM.pdf]

Supplementary Figure 4

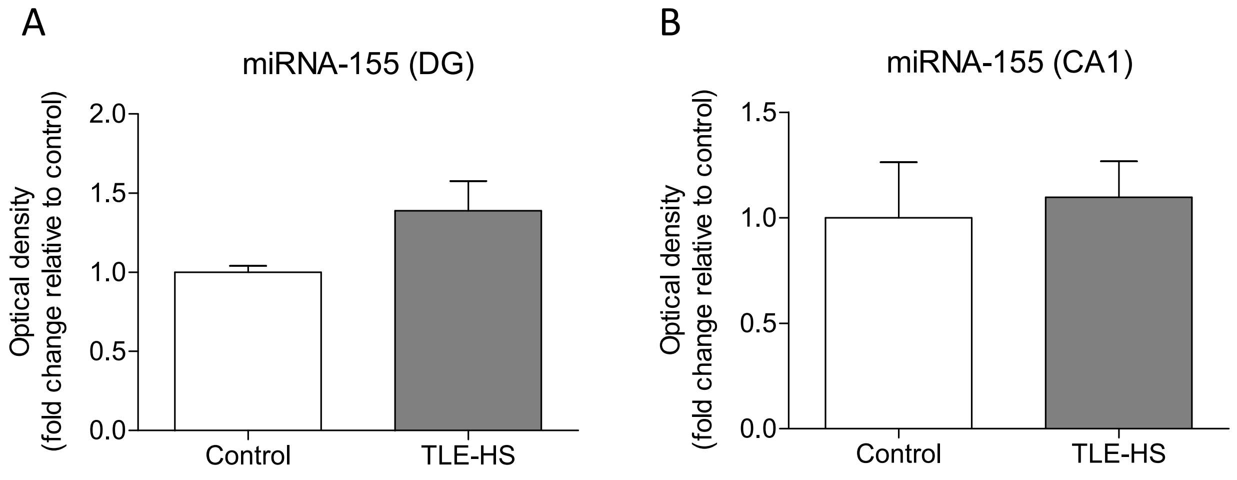

Supplementary Fig. 4. Quantitative analysis of miR-155 in situ hybridization in the dentate gyrus (DG, **A**) ot CA1 area (**B**)of resected brain tissue of patients with TLE-HS showed a trend towards upregulation in the DG. Mann-Whitney U test, error bars depict the standard error of the mean.
