# Supplementary material for: Increased expression of matrix metalloproteinase 3 can be attenuated by inhibition of microRNA-155 in cultured human astrocytes
Source: J Neuroinflammation. 2018 Jul 21;15:211. doi: 10.1186/s12974-018-1245-y (PMC6054845; doi:10.1186/s12974-018-1245-y)
Supplement: Supplementary file 6 — Figure S5. Densitometrical quantification of miR-155 expression in the rat TLE model. (PDF 89 kb) [file 12974_2018_1245_MOESM6_ESM.pdf]

Supplementary Figure 5

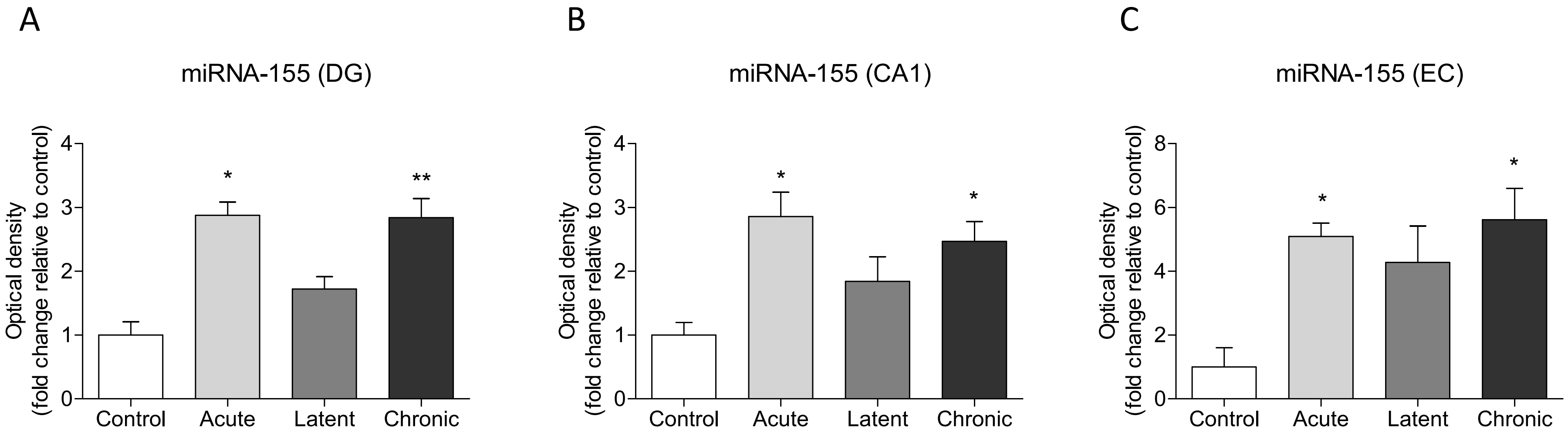

Supplementary Fig. 5. Quantitative analysis of miR-155 in situ hybridization in the rat TLE model showed increased miR-155 expression in the dentate gyrus (DG; **A**, acute stage $p<0.05$, chronic stage $p<0.01$), CA1 area (**B,** acute stage $p<0.05$, chronic stage $p<0.05$), and entorhinal cortex (EC) (**C**, acute stage $p<0.05$, chronic stage $p<0.01$); Mann-Whitney U test, error bars depict the standard error of the mean.
